# Musculoskeletal involvement in sarcoidosis*, **

**DOI:** 10.1590/S1806-37132014000200012

**Published:** 2014

**Authors:** Akasbi Nessrine, Abourazzak Fatima Zahra, Harzy Taoufik

**Affiliations:** Hassan II University Hospital, Fez, Morocco; Hassan II University Hospital, Fez, Morocco; Hassan II University Hospital, Fez, Morocco

**Keywords:** Sarcoidosis, Joints, Muscles, Bone and Bones

## Abstract

Sarcoidosis is a multisystem inflammatory disorder of unknown cause. It most commonly affects the pulmonary system but can also affect the musculoskeletal system, albeit less frequently. In patients with sarcoidosis, rheumatic involvement is polymorphic. It can be the presenting symptom of the disease or can appear during its progression. Articular involvement is dominated by nonspecific arthralgia, polyarthritis, and Löfgren's syndrome, which is defined as the presence of lung adenopathy, arthralgia (or arthritis), and erythema nodosum. Skeletal manifestations, especially dactylitis, appear mainly as complications of chronic, multiorgan sarcoidosis. Muscle involvement in sarcoidosis is rare and usually asymptomatic. The diagnosis of rheumatic sarcoidosis is based on X-ray findings and magnetic resonance imaging findings, although the definitive diagnosis is made by anatomopathological study of biopsy samples. Musculoskeletal involvement in sarcoidosis is generally relieved with nonsteroidal anti-inflammatory drugs or corticosteroids. In corticosteroid-resistant or -dependent forms of the disease, immunosuppressive therapy, such as treatment with methotrexate or anti-TNF-α, is employed. The aim of this review was to present an overview of the various types of osteoarticular and muscle involvement in sarcoidosis, focusing on their diagnosis and management.

## Introduction

Sarcoidosis is a granulomatous disease of unknown etiology that involves multiple systems. It most commonly affects the lungs, lymph nodes, skin, and eyes but can also affect other organs and systems, including the musculoskeletal system.^(^ ^1^ ^)^ Rheumatic manifestations of sarcoidosis, although rare, include inflammatory arthritis, periarticular soft tissue swelling, tenosynovitis, dactylitis, bone involvement, sarcoid myopathy, and bone loss. The primary types of articular involvement are Löfgren's syndrome and acute polyarthritis, whereas bone involvement is dominated by sarcoid dactylitis and osteolysis. Muscle involvement often goes unrecognized and can appear as chronic myopathy, acute myositis, or pseudotumor. Sarcoidosis can also manifest as calcium balance disturbances, principally hypercalcemia, which is often asymptomatic but can occasionally be the presenting clinical symptom of the sarcoidosis.^(^ ^2^ ^)^

The diagnosis of sarcoidosis is based on clinical and radiological findings, together with evidence of noncaseating granulomas in biopsy specimens, after other granulomatous disorders, such as tuberculosis, have been excluded.^(^ ^2^ ^)^ Treatment of rheumatic involvement often requires the use of nonsteroidal anti-inflammatory drugs (NSAIDs), corticosteroids, or methotrexate. Biological therapies such as the use of anti-TNF-α agents and anti-CD20 monoclonal antibodies have been shown to be effective in some cases of severe or refractory sarcoidosis.^(^ ^3^ ^)^ The aim of this review was to present an overview of the various types of musculoskeletal involvement in sarcoidosis, focusing on their diagnosis and management.

## Physiopathology

The exact cause of sarcoidosis remains unknown. The Th1-type of inflammation is present in the sarcoid granuloma which expresses and produces a variety of inflammatory cytokines, such as IL-2, IL-12, IL-6, and IFN-γ, as well as TNF-α, which is the central mediator of this inflammatory process.^(^ ^4^ ^)^ Because of clinical and histological similarities with mycobacterial and fungal diseases, infectious causes have been investigated. However, such studies are controversial.^(^ ^5^ ^)^ Recent evidence suggests that a genetic component is implicated in susceptibility to sarcoidosis. There is a strong link between sarcoidosis and variants in the class I and II HLA locus. A recent study identified annexin A11 as a novel non-HLA susceptibility locus for sarcoidosis.^(^ ^6^ ^)^ Many other loci encoding TNF-α and co-stimulatory molecules on antigen-presenting cells such as CD80 and CD86, as well as the chemokine receptors CCR2 and CCR5, have been found to increase susceptibility to sarcoidosis.^(^ ^7^ ^)^

## Articular involvement

The reported prevalence of arthritis in sarcoidosis ranges from 10% to 38%.^(^ ^8^ ^)^ Nonspecific arthralgia affects the majority of sarcoidosis patients, especially females. With the exception of Löfgren's syndrome, joint manifestations are rarely seen at symptom onset in sarcoidosis. Two types of arthritis, differing in their clinical course and prognosis, have been identified. The first is acute polyarthritis, which is typically accompanied by erythema nodosum and occasionally by acute uveitis. Acute polyarthritis resolves without permanent sequelae. The second type is chronic sarcoid arthritis, which, although less common, can progress to joint deformity. Other forms of articular manifestations, such as periarticular soft tissue swelling and tenosynovitis, can also be seen.^(^ ^9^ ^)^

### Acute arthropathy

Acute polyarthritis occurs in 40% of patients with sarcoidosis, particularly in the earlier stages of the disease, and can be the presenting feature. It is self-limiting, is usually symmetric, and resolves without permanent sequelae.^(^ ^10^ ^)^ The most common form of acute arthropathy in sarcoidosis is Löfgren's syndrome, which occurs in acute onset sarcoidosis and typically manifests as bilateral hilar lymphadenopathy, arthritis, and erythema nodosum. Löfgren's syndrome is associated with a good prognosis and spontaneous remission.^(^ ^11^ ^,^ ^12^ ^)^ In patients with sarcoidosis, acute polyarthritis most commonly involves the ankles (in > 90% of cases), often bilaterally, followed by other large joints of the lower limbs, only occasionally involving the small joints of hands and feet. This type of polyarthritis is only mildly painful, migratory and transient. Oligoarthritis or monoarthritis are relatively rare forms of acute sarcoid arthropathy.

### Chronic arthropathy

Chronic arthropathy is rare in sarcoidosis, occurring in only 0.2% of cases. It most often affects black males and is usually accompanied by other systemic disorders, mainly those of the lungs and eyes.^(^ ^4^ ^)^ Various forms of chronic arthritis can occur in patients with sarcoidosis: nondeforming arthritis with granulomatous synovitis; Jaccoud's arthropathy; and joint swelling adjacent to a sarcoid bone lesion. Among such patients, the arthritis is rheumatoid factor-positive in 10-47% of cases. That nonspecific reactivity is due to increased circulating polyclonal IgG. Therefore, sarcoidosis-related arthritis can mimic rheumatoid arthritis, especially when accompanied by joint deformities. The differential diagnosis is usually made on the basis of clinical criteria, including negative serology for anti-cyclic citrullinated peptide antibodies and antinuclear antibodies, as well as the absence of the specific erosive joint deformity seen in rheumatoid arthritis. ^(^ ^13^ ^)^ A finding of granuloma on synovial biopsy helps in establishing the diagnosis of sarcoidosis. Although all joints can be affected, affected ankles strongly indicate the diagnosis of sarcoidosis. In some cases, X-rays show soft tissue swelling. Magnetic resonance imaging (MRI) can depict lesions that cannot be visualized on X-rays.

### Involvement of periarticular structures

Among patients with sarcoidosis, tenosynovitis is common in the tendons of the ankles and wrist, occasionally accompanied by carpal tunnel syndrome in the latter case.^(^ ^14^ ^)^ Although tenosynovitis, tendinitis, bursitis, and synovitis can be demonstrated on MRI scans, they are nonspecific findings and biopsy is therefore required in order to confirm the diagnosis of sarcoidosis.^(^ ^15^ ^)^

### Sacroiliitis in sarcoidosis

Sacroiliac involvement in sarcoidosis is rare and generally unilateral. Sarcoidosis cannot be established without a biopsy to rule out tuberculosis or other infectious process of that joint.^(^ ^16^ ^)^ Sacroiliitis can reveal ankylosing spondylitis that can be associated with sarcoidosis, especially in patients testing positive for HLA-B27.

### Treatment of sarcoid arthropathy

In 90% of cases, acute polyarthritis resolves spontaneously. In others, it requires treatment with NSAIDs, corticosteroid injections into the joint, or a short course of corticosteroids at 10-15 mg/day. Hydroxychloroquine and colchicine can be used in some cases, especially in those of Löfgren's syndrome.^(^ ^17^ ^)^ The use of other immunosuppressive agents should be reserved for patients with progressive chronic sarcoid arthropathy that is refractory to treatment with systemic corticosteroids or in whom steroids have generated side effects. According to the Brazilian Thoracic Association Guidelines for Interstitial Lung Diseases,^(^ ^18^ ^)^ there are alternative treatments, such as methotrexate, azathioprine, leflunomide and hydroxychloroquine. Methotrexate is an efficient and corticosteroid-sparing therapeutic agent for the treatment of musculoskeletal manifestations of sarcoidosis.^(^ ^19^ ^)^ Many studies have shown the importance of TNF-α in sarcoid granuloma development, which makes TNF-α a potential target in the treatment of sarcoidosis. Many interesting reports suggest some efficacy of TNF-α antagonists (infliximab, etanercept, and adalimumab) in refractory sarcoidosis with musculoskeletal involvement.^(^ ^20^ ^-^ ^23^ ^)^ Paradoxical cases of proven sarcoidosis have been reported in patients receiving anti-TNF-α agents for other chronic inflammatory rheumatic diseases. This paradoxical effect of anti-TNF-α agents must be known by the clinician.^(^ ^24^ ^,^ ^25^ ^)^ The use of B-cell-depleting agents might also be of benefit in sarcoid arthritis. A recent case report of a patient with sarcoidosis of the lungs and joints showed that rituximab is effective in treating sarcoidosis without major side effects.^(^ ^26^ ^)^ Based on the success of rituximab in this disease, other B-cell therapies, such as ocrelizumab, need to be evaluated in systemic sarcoidosis.^(^ ^27^ ^)^

## Osseous sarcoidosis

Bone involvement is reported in 1-15% of sarcoidosis patients. It is more common in black patients and is usually accompanied by infiltrative skin lesions, especially lupus pernio.^(^ ^28^ ^)^

### Involvement of small bones

Although bone lesions are frequently asymptomatic, some sarcoidosis patients present with symptomatic dactylitis. Bone, skin and soft tissue are involved, especially in the second and third phalanges, resulting in sausage-like fingers resembling those seen in the spondyloarthropathies. ^(^ ^29^ ^)^

The bony lesions are usually cystic, sclerotic lesions rarely being reported. Multiple cystic lesions sometimes result in a "lacy" pattern, which is typical of sarcoid bone disease. The classic lesions in the small bones of the hands and feet are known as Perthes disease and Jüngling's disease. They are well characterized on standard X-rays.

**Figure 1:**
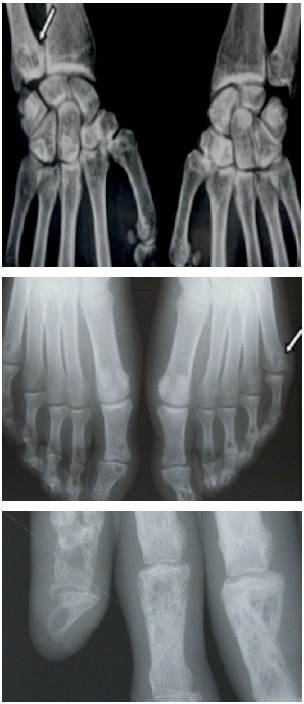
Pattern of bone involvement found on X-rays in a patient with sarcoidosis: multiple, large cysts (type I). From the collection of Professor Yannick Allanore, of the Department of Rheumatology A, Descartes University, Medical School, Cochin Hospital, Paris, France. Used with the permission of Professor Allanore.

**Figure 2:**
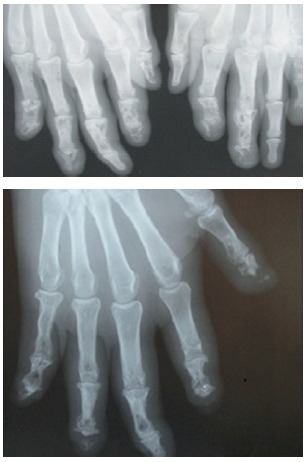
X-ray of hands showing cysts and acro-osteolysis (type II). From the collection of Professor Yannick Allanore, of the Department of Rheumatology A, Descartes University, Medical School, Cochin Hospital, Paris, France. Used with the permission of Professor Allanore.

There are three radiological types of sarcoid bone disease: type I, characterized by big cystic lesions (Figure 1), which is quite rare and can be associated with a stress fracture from a pathological fracture; type II, characterized by multiple, small circumscribed cysts, occasionally conflicting, and polycyclic (Figure 2); and type III, characterized by tunneling of the cortex of the phalanx, which leads to remodeling of the cortical and trabecular architecture. All three forms can coexist in the same bone. Acro-osteolysis, presenting as nodular densities in the terminal phalanges, can also occur.^(^ ^17^ ^)^

### Involvement of long bones

Involvement of the axial skeleton and long bones is uncommon in sarcoidosis. Vertebral sarcoidosis can present as purely lytic lesions, as purely sclerotic lesions (in rare cases) mimicking blastic metastases, or as a mixture of the two. The lower dorsal and upper lumbar vertebrae are mostly involved. Because it can guide the selection of biopsy sites, MRI has gained attention as a modality that facilitates the histopathological confirmation of the diagnosis and can be used in evaluating the efficacy of the treatment of bone lesions. The MRI findings are nonspecific; showing multifocal lesions that are hypointense on T1-weighted images and hyperintense on T2-weighted images.^(^ ^30^ ^)^

Any bone, including the skull, ribs, nasal bone, and long bones, can be affected in sarcoidosis. In the skull, sarcoidosis manifests as asymmetrical, asymptomatic lytic lesions of variable size. Positron emission tomography/computed tomography (PET/CT) imaging can be useful in the assessment of bone involvement in sarcoidosis patients.^(^ ^31^ ^)^

### Treatment of osseous sarcoidosis

Asymptomatic osseous sarcoidosis generally does not require therapy, although the indications for therapeutic intervention are not well defined. However, treatment is usually indicated when the symptoms include uncontrolled pain, stiffness, or bony destruction. Therapy generally consists of oral corticosteroids at 15-20 mg/day. The dosage is adjusted according to the clinical response.^(^ ^32^ ^)^ Methotrexate and hydroxychloroquine can also be used. Although there is some evidence that anti-TNF-α agents are efficacious in sarcoid bone lesions, this effect needs to be verified.^(^ ^33^ ^)^

## Muscle sarcoidosis

Muscle sarcoidosis is a rare entity and is usually asymptomatic. It is symptomatic in only 1% of cases. It typically appears as a complication of systemic sarcoidosis.^(^ ^34^ ^)^ The histological pattern of sarcoid myopathy is as a noncaseating granuloma in the perimysial connective tissue. Large granulomas compress and destroy adjacent muscle fibers, resulting in degeneration and focal lymphocyte infiltration, and foci of necrosis or fibrosis can also be observed.^(^ ^34^ ^)^ Fiber destruction in this disease is caused mainly by fiber infiltration rather than by mechanical compression or ischemia.

In a patient with sarcoidosis, the presence of muscle weakness, muscle pain, or muscle nodules is suggestive of sarcoid myopathy. Fatigue and general weakness are common, which could explain why patients with sarcoidosis frequently experience exercise intolerance.^(^ ^35^ ^,^ ^36^ ^)^ The cause is not only sarcoid myopathy but also the high circulating levels of inflammatory cytokines such as TNF-α, IL-6, and IFN-γ.

In sarcoid myopathy, three clinical patterns are generally recognized^(^ ^37^ ^)^: chronic myopathy (seen in 86% of cases), which is the most common form, characterized by an insidious onset of proximal muscle weakness with normal or elevated serum levels of muscle enzymes; acute myositis (seen in 11% of cases); and nodular or tumor-like myositis (seen in only 3% of cases). Nodular myopathy manifests as multiple, tumor-like, palpable nodules in the muscles.^(^ ^37^ ^,^ ^38^ ^)^ The use of MRI and PET/CT facilitates the diagnosis of muscle sarcoidosis.

The mainstay of the treatment of patients with muscle sarcoidosis is 8-12 weeks of systemic glucocorticoid therapy at an initial daily dose of 0.5-1 mg/kg with progressive tapering. Methotrexate, chloroquine and azathioprine have been used in corticosteroid-resistant and corticosteroid-dependent forms.^(^ ^39^ ^)^ Thalidomide and infliximab have been found to be beneficial in some cases of sarcoid myopathy. The effectiveness of these medications seems related to TNF-α inhibition. Corticosteroid-induced myopathy can also occur as a complication of the treatment of sarcoidosis. Affected patients typically develop proximal muscle weakness that has a gradual onset (over several weeks) and is accompanied by muscle wasting. A common manifestation is difficulty getting up from a chair or climbing stairs. Myalgia and muscle tenderness are not observed.^(^ ^40^ ^)^

## Combination of sarcoidosis and rheumatic disease

Sarcoidosis can be associated with other chronic inflammatory disease like systemic lupus erythematosus (SLE), Sjögren's syndrome or psoriatic arthritis. In sarcoidosis, suspicion of SLE is raised when the patient develops a butterfly rash or discoid lesions. The treatment of sarcoidosis patients with SLE is challenging and should be individualized.^(^ ^41^ ^)^ The use of anti-TNF-α agents should be avoided in patients who have active SLE. In rare cases, sarcoidosis and Sjögren's syndrome can both affect the salivary glands. Dryness and diffuse swelling of oral mucosal tissues can be the presenting symptom of sarcoidosis.^(^ ^42^ ^)^

Approximately 6% of all patients with sarcoidosis develop a psoriatic form of arthritis. ^(^ ^43^ ^)^ Although anti-TNF-α therapy is helpful in psoriasis, it can also paradoxically induce progressive psoriasis, and patients treated with anti-TNF-α agents should be closely monitored.

## Changes in calcium metabolism

In sarcoidosis, hypercalciuria is more common than is hypercalcemia. Either can be caused by nephrocalcinosis, kidney stones, or renal failure. Granulomatous macrophages increase conversion of 25-hydroxyvitamin D to active 1,25-dihydroxyvitamin D (calcitriol), leading to increased calcium absorption by the intestine. ^(^ ^44^ ^)^ The high levels of calcitriol induce osteoclast activation and bone resorption. In addition, corticosteroid-treated patients are at a higher risk of osteoporosis. Bone loss could be also increased by high levels of parathyroid hormone-related peptide identified in sarcoid tissue.^(^ ^45^ ^)^ Corticosteroids have been successfully used to improve disorders of calcium metabolism. ^(^ ^46^ ^)^ Mycophenolate mofetil and infliximab have been used in select cases.^(^ ^47^ ^,^ ^48^ ^)^

## Bone loss in sarcoidosis

The bone loss in sarcoidosis can be caused by multiple factors, including diffuse skeletal granulomatosis, calcitriol, osteoclast activating factor, and glucocorticoid therapy, particularly in postmenopausal patients. In one study of corticosteroid therapy in patients with sarcoidosis, the authors found that the rate of bone loss in corticosteroid-treated postmenopausal patients with sarcoidosis was greater than that reported for corticosteroid-treated patients with rheumatoid arthritis or asthma.^(^ ^49^ ^)^

The prevention and treatment of bone loss in patients with sarcoidosis is difficult. Calcium and vitamin D, both commonly administered to patients at risk for osteoporosis, should be considered with caution in patients with hypercalcemia, hypercalciuria, high levels of parathormone, and kidney stones.^(^ ^50^ ^)^ Guidelines published by the American College of Rheumatology recommend that patients receiving corticosteroids should undergo bone mineral density testing.^(^ ^51^ ^)^ The World Health Organization fracture assessment risk tool can be used in order to calculate patient risk of fracture.

For patients with sarcoidosis at risk of developing osteoporosis because of prolonged use of corticosteroids, bisphosphonates are effective in preventing glucocorticoid-induced bone loss. The American College of Rheumatology recommends using bisphosphonates in low-risk patients receiving corticosteroids at doses of = 7.5 mg/day, as well as in all medium- or high-risk patients receiving corticosteroids. Of the available bisphosphonates, alendronate, risedronate, zoledronic acid, and teriparatide effectively reduce bone loss and can thus diminish fracture risk.^(^ ^51^ ^)^ Further studies on bisphosphonate use in osteoporosis during sarcoidosis are needed.

## Final considerations

Although sarcoidosis can affect any organ, sarcoidosis involving the musculoskeletal system is rare. The disease can affect the muscles, joints and the bones. Those conditions, which are polymorphic, can be the presenting symptoms the disease or can appear during the course of its progression. Corticosteroids are the cornerstone of sarcoidosis treatment but only have a postponing effect. Prospective, randomized, controlled trials assessing anti-TNF-α agents are needed in order to evaluate their efficacy in cases of sarcoidosis with rheumatic complications.
