# Supplementary material for: Musculoskeletal involvement in sarcoidosis
Source: J Bras Pneumol. 2014 Mar-Apr;40(2):175–82. doi: 10.1590/S1806-37132014000200012 (PMC4083650; doi:10.1590/S1806-37132014000200012)
Supplement: Translation

# Translation: Acometimento músculo-esquelético na sarcoidose*

**Authors:** Akasbi Nessrine, Abourazzak Fatima Zahra, Harzy Taoufik

**Affiliations:** Hospital Universitário Hassan II, Fez, Marrocos; Hospital Universitário Hassan II, Fez, Marrocos; Hospital Universitário Hassan II, Fez, Marrocos

**Keywords:** Sarcoidose, Articulações, Músculos, Osso e Ossos

## Abstract

A sarcoidose é um distúrbio inflamatório multissistêmico de causa desconhecida, frequentemente afetando o sistema pulmonar e também o sistema músculo-esquelético, mas de forma menos frequente. Em pacientes com sarcoidose, o acometimento reumático é polimórfico, podendo ser o sintoma de apresentação da doença ou aparecer durante sua progressão. O acometimento articular é dominado por artralgia inespecífica, poliartrite e síndrome de Löfgren, que é definida como a presença de adenopatia pulmonar, artralgia (ou artrite) e eritema nodoso. Manifestações esqueléticas, especialmente dactilite, aparecem principalmente como complicações de sarcoidose crônica e em vários órgãos. O acometimento muscular na sarcoidose é raro e geralmente assintomático. O diagnóstico de sarcoidose reumática baseia-se em achados radiográficos e de ressonância magnética, embora o diagnóstico definitivo seja feito pelo estudo anatomopatológico de amostras de biópsia. O acometimento músculo-esquelético na sarcoidose é geralmente aliviado com o uso de anti-inflamatórios não esteroidais ou corticosteroides. Em formas da doença resistentes ao corticosteroide ou corticosteroide dependentes, a terapia de imunossupressão, como o tratamento com metotrexato ou anti-TNF-α, é utilizada. O objetivo desta revisão foi apresentar uma visão geral dos vários tipos de acometimento osteoarticular e muscular na sarcoidose, com foco no diagnóstico e manejo.

## Introdução

A sarcoidose é uma doença granulomatosa de etiologia desconhecida que acomete vários sistemas. Afeta mais frequentemente pulmões, linfonodos, pele e olhos, mas também pode afetar outros órgãos e sistemas, incluindo o sistema músculo-esquelético.^(^ ^1^ ^)^ As manifestações reumáticas da sarcoidose, embora raras, incluem artrite inflamatória, edema de partes moles periarticulares, tenossinovite, dactilite, acometimento ósseo, miopatia sarcoide e perda óssea. Os principais tipos de acometimento articular são síndrome de Löfgren e poliartrite aguda, enquanto o acometimento ósseo é dominado por dactilite sarcoide e osteólise. O acometimento muscular muitas vezes passa despercebido, podendo aparecer como miopatia crônica, miosite aguda ou pseudotumor. A sarcoidose também pode se manifestar como distúrbios no equilíbrio de cálcio, principalmente hipercalcemia, que muitas vezes é assintomática, mas pode ocasionalmente ser o sintoma clínico de apresentação da sarcoidose.^(^ ^2^ ^)^

O diagnóstico de sarcoidose baseia-se em achados clínicos e radiológicos, juntamente com evidências de granulomas não caseosos em espécimes de biópsia, após outros distúrbios granulomatosos, como a tuberculose, terem sido excluídos.^(^ ^2^ ^)^ O tratamento do acometimento reumático muitas vezes requer o uso de anti-inflamatórios não esteroidais (AINEs), corticosteroides ou metotrexato. As terapias biológicas como o uso de agentes anti-TNF-α e anticorpos monoclonais anti-CD20 têm se mostrado eficazes em alguns casos de sarcoidose grave ou refratária.^(^ ^3^ ^)^ O objetivo desta revisão foi apresentar uma visão geral dos vários tipos de acometimento músculo-esquelético na sarcoidose, com foco no diagnóstico e manejo.

## Fisiopatologia

A causa exata da sarcoidose permanece desconhecida. A inflamação do tipo Th1 está presente no granuloma sarcoide que expressa e produz uma variedade de citocinas inflamatórias, como IL-2, IL-12, IL-6 e IFN-γ, assim como TNF-α, que é o mediador central desse processo inflamatório.^(^ ^4^ ^)^ Em virtude de semelhanças clínicas e histológicas com doenças micobacterianas e fúngicas, causas infecciosas têm sido investigadas. Porém, tais estudos são controversos.^(^ ^5^ ^)^ Evidências recentes sugerem que um componente genético está implicado na suscetibilidade à sarcoidose. Há uma forte ligação entre a sarcoidose e variantes no lócus HLA de classe I e II. Um estudo recente identificou a anexina A11 como um novo lócus não-HLA de suscetibilidade para a sarcoidose.^(^ ^6^ ^)^ Muitos outros locos que codificam TNF-α e moléculas coestimuladoras em células apresentadoras de antígenos como CD80 e CD86, assim como os receptores de quimiocinas CCR2 e CCR5, mostraram aumentar a suscetibilidade à sarcoidose.^(^ ^7^ ^)^

## Acometimento articular

A prevalência relatada de artrite na sarcoidose varia de 10% a 38%.^(^ ^8^ ^)^ A artralgia inespecífica afeta a maioria dos pacientes com sarcoidose, especialmente os do sexo feminino. Excetuando-se a síndrome de Löfgren, manifestações articulares são raramente vistas no início dos sintomas na sarcoidose. Dois tipos de artrite, que diferem quanto à evolução clínica e ao prognóstico, foram identificados. O primeiro é a poliartrite aguda, que é tipicamente acompanhada de eritema nodoso e ocasionalmente de uveíte aguda. A poliartrite aguda se resolve sem sequelas permanentes. O segundo tipo é a artrite sarcoide crônica, que, embora menos frequente, pode evoluir para deformidade articular. Outras formas de manifestações articulares, como o edema de partes moles periarticulares e a tenossinovite, também podem ser vistas.^(^ ^9^ ^)^

### Artropatia aguda

A poliartrite aguda ocorre em 40% dos pacientes com sarcoidose, particularmente nos estágios iniciais da doença, e pode ser a característica de apresentação. É autolimitada, geralmente simétrica e se resolve sem sequelas permanentes.^(^ ^10^ ^)^ A forma mais frequente de artropatia acuda na sarcoidose é a síndrome de Löfgren, que ocorre na sarcoidose de início agudo e tipicamente se manifesta como linfadenopatia hilar bilateral, artrite e eritema nodoso. A síndrome de Löfgren está associada a bom prognóstico e remissão espontânea.^(^ ^11^ ^,^ ^12^ ^)^ Em pacientes com sarcoidose, a poliartrite aguda acomete mais frequentemente os tornozelos (em > 90% dos casos), muitas vezes bilateralmente, seguidos de outras grandes articulações dos membros inferiores, acometendo apenas ocasionalmente as pequenas articulações das mãos e dos pés. Esse tipo de poliartrite é apenas levemente doloroso, migratório e transitório. Oligoartrite ou monoartrite são formas relativamente raras de artropatia sarcoide aguda.

### Artropatia crônica

A artropatia crônica é rara na sarcoidose, ocorrendo em apenas 0,2% dos casos. Afeta mais frequentemente indivíduos negros do sexo masculino e é geralmente acompanhada de distúrbios sistêmicos, principalmente os distúrbios dos pulmões e dos olhos.^(^ ^4^ ^)^ Várias formas de artrite crônica podem ocorrer em pacientes com sarcoidose: artrite não deformadora com sinovite granulomatosa; artropatia de Jaccoud; e edema articular adjacente a uma lesão sarcoide do osso. Entre tais pacientes, a artrite é positiva para fator reumatoide em 10-47% dos casos. Essa reatividade inespecífica é devida ao aumento de IgG policlonal circulante. Portanto, a artrite por sarcoidose pode simular a artrite reumatoide, especialmente quando acompanhada de deformidades articulares. O diagnóstico diferencial geralmente é feito com base em critérios clínicos, incluindo sorologia negativa para anticorpos anti-peptídeos citrulinados cíclicos e para anticorpos antinucleares, assim como a ausência da deformidade articular erosiva específica vista na artrite reumatoide.^(^ ^13^ ^)^ O achado de granuloma na biópsia sinovial ajuda a estabelecer o diagnóstico de sarcoidose. Embora todas as articulações possam ser afetadas, tornozelos afetados indicam fortemente o diagnóstico de sarcoidose. Em alguns casos, as radiografias mostram edema de partes moles. A ressonância magnética (RM) é capaz de revelar lesões que não podem ser visualizadas nas radiografias.

### Acometimento de estruturas periarticulares

Entre os pacientes com sarcoidose, a tenossinovite é frequente nos tendões dos tornozelos e pulsos, ocasionalmente acompanhada de síndrome do túnel do carpo no segundo caso. ^(^ ^14^ ^)^ Embora a tenossinovite, a tendinite, a bursite e a sinovite possam ser demonstradas em imagens de RM, são achados inespecíficos, e, portanto, a biópsia é necessária para a confirmação do diagnóstico de sarcoidose.^(^ ^15^ ^)^

### Sacroileíte na sarcoidose

O acometimento sacroilíaco na sarcoidose é raro e geralmente unilateral. A sarcoidose não pode ser estabelecida sem uma biópsia para excluir a tuberculose ou outros processos infecciosos dessa articulação.^(^ ^16^ ^)^ A sacroileíte pode revelar espondilite anquilosante que pode estar associada à sarcoidose, especialmente em pacientes HLA-B27-positivos.

### Tratamento da artropatia sarcoide

Em 90% dos casos, a poliartrite aguda se resolve espontaneamente. Em outros, requer tratamento com AINEs, injeções de corticosteroides na articulação ou um curso curto de 10-15 mg/dia de corticosteroides. A hidroxicloroquina e a colchicina podem ser usadas em alguns casos, especialmente nos de síndrome de Löfgren.^(^ ^17^ ^)^ O uso de outros agentes imunossupressores deve ser reservado para pacientes com artropatia sarcoide crônica progressiva refratária a tratamento com corticosteroides sistêmicos ou nos quais os esteroides geraram efeitos colaterais. De acordo com as Diretrizes de Doenças Pulmonares Intersticiais da Sociedade Brasileira de Pneumologia e Tisiologia,^(^ ^18^ ^)^ existem tratamentos alternativos, como o metotrexato, a azatioprina, a leflunomida e a hidroxicloroquina. O metotrexato é um agente terapêutico eficiente e poupador de corticosteroides para o tratamento de manifestações músculo-esqueléticas da sarcoidose.^(^ ^19^ ^)^ Muitos estudos demonstraram a importância do TNF-α no desenvolvimento do granuloma sarcoide, o que faz do TNF-α um potencial alvo no tratamento da sarcoidose. Muitos relatos interessantes sugerem alguma eficácia de antagonistas do TNF-α (infliximabe, etanercepte e adalimumabe) na sarcoidose refratária com acometimento músculo-esquelético.^(^ ^20^ ^-^ ^23^ ^)^ Casos paradoxais de sarcoidose comprovada foram relatados em pacientes tratados com agentes anti-TNF-α para outras doenças reumáticas inflamatórias crônicas. Esse efeito paradoxal dos agentes anti-TNF-α deve ser conhecido pelo clínico.^(^ ^24^ ^,^ ^25^ ^)^ É possível que o uso de agentes depletores de células B também possa ser benéfico na artrite sarcoide. Um relato de caso recente de um paciente com sarcoidose dos pulmões e articulações demonstrou que o rituximabe é efetivo no tratamento da sarcoidose, sem grandes efeitos colaterais.^(^ ^26^ ^)^ Com base no sucesso do rituximabe nessa doença, outras terapias com alvo em células B, como o ocrelizumabe, precisam ser avaliadas na sarcoidose sistêmica.^(^ ^27^ ^)^

## Sarcoidose óssea

O acometimento ósseo é relatado em 1-15% dos pacientes com sarcoidose. É mais frequente em pacientes negros e é geralmente acompanhado de lesões cutâneas infiltrativas, especialmente o lúpus pernio.^(^ ^28^ ^)^

### Acometimento de pequenos ossos

Embora as lesões ósseas sejam frequentemente assintomáticas, alguns pacientes com sarcoidose apresentam dactilite sintomática. Osso, pele e tecido mole estão envolvidos, especialmente na segunda e terceira falanges, resultando em dedos em salsicha que lembram aqueles vistos nas espondiloartropatias.^(^ ^29^ ^)^

As lesões ósseas geralmente são císticas, sendo raros os relatos de lesões escleróticas. Múltiplas lesões císticas às vezes resultam em um padrão "rendado", que é típico da doença sarcoide do osso. As lesões clássicas nos pequenos ossos das mãos e dos pés são conhecidas como doença de Perthes e doença de Jüngling. São bem caracterizadas nas radiografias padrão.

Existem três tipos radiológicos de doença sarcoide do osso: tipo I, caracterizado por grandes lesões císticas (Figura 1), que é bastante raro e pode estar associado a uma fratura por estresse a partir de uma fratura patológica; tipo II, caracterizado por múltiplos pequenos cistos circunscritos, ocasionalmente conflitantes, e policíclicos (Figura 2); e tipo III, caracterizado por tunelização da cortical da falange, que leva ao remodelamento da arquitetura cortical e trabecular. Todas as três formas podem coexistir no mesmo osso. Também pode ocorrer acro-osteólise, que se apresenta como densidades nodulares nas falanges terminais.^(^ ^17^ ^)^

**Figura 1:** Padrão de acometimento ósseo encontrado em radiografias de paciente com sarcoidose: múltiplos cistos grandes (tipo I). Da coleção do Professor Yannick Allanore, do Departamento de Reumatologia A, Universidade Descartes, Faculdade de Medicina, Hospital Cochin, Paris, França. Usadas com permissão do Professor Allanore.

**Figura 2:** Radiografias de mão evidenciando cistos e acro-osteólise (tipo II). Da coleção do Professor Yannick Allanore, do Departamento de Reumatologia A, Universidade Descartes, Faculdade de Medicina, Hospital Cochin, Paris, França. Usadas com permissão do Professor Allanore.

### Acometimento de ossos longos

O acometimento do esqueleto axial e de ossos longos é infrequente na sarcoidose. A sarcoidose vertebral pode se apresentar como lesões puramente líticas, como lesões puramente escleróticas (em casos raros) simulando metástases blásticas ou como uma mistura das duas. As vértebras dorsais inferiores e lombares superiores são acometidas em sua maioria. Uma vez que a RM pode orientar a seleção de locais de biópsia, ela tem recebido atenção como modalidade facilitadora da confirmação histopatológica do diagnóstico e pode ser usada na avaliação da eficácia do tratamento das lesões ósseas. Os achados da RM são inespecíficos, mostrando lesões multifocais que são hipointensas em imagens em T1 e hiperintensas em imagens em T2.^(^ ^30^ ^)^

Qualquer osso, incluindo o crânio, as costelas, o osso nasal e os ossos longos, pode ser afetado na sarcoidose. No crânio, a sarcoidose se manifesta como lesões líticas assimétricas assintomáticas de tamanho variado.

Imagens de* positron emission tomography*/tomografia computadorizada (PET/TC) podem ser úteis na avaliação de acometimento ósseo em pacientes com sarcoidose.^(^ ^31^ ^)^

### Tratamento da sarcoidose óssea

A sarcoidose óssea assintomática em geral não requer terapia, embora as indicações para intervenção terapêutica não estejam bem definidas. Porém, geralmente se indica tratamento quando os sintomas incluem dor não controlada, rigidez ou destruição óssea. A terapia em geral consiste em 15-20 mg/dia de corticosteroides orais. A dosagem é ajustada de acordo com a resposta clínica.^(^ ^32^ ^)^ O metotrexato e a hidroxicloroquina também podem ser usados. Embora existam evidências de que os agentes anti-TNF-α são eficazes nas lesões sarcoides do osso, esse efeito precisa ser verificado.^(^ ^33^ ^)^

## Sarcoidose muscular

A sarcoidose muscular é uma entidade rara e geralmente assintomática. É sintomática em apenas 1% dos casos. Tipicamente aparece como complicação da sarcoidose sistêmica.^(^ ^34^ ^)^ O padrão histológico da miopatia sarcoide é o de granuloma não caseoso no tecido conjuntivo perimisial. Grandes granulomas comprimem e destroem as fibras musculares adjacentes, resultando em degeneração e infiltração linfocitária focal, e focos de necrose ou fibrose também podem ser observados.^(^ ^34^ ^)^ A destruição das fibras nessa doença é causada principalmente por infiltração das fibras, em vez de por compressão mecânica ou isquemia.

Em um paciente com sarcoidose, a presença de fraqueza muscular, dor muscular ou nódulos musculares é sugestiva de miopatia sarcoide. Fatiga e fraqueza geral são comuns, o que poderia explicar porque pacientes com sarcoidose frequentemente apresentam intolerância ao exercício.^(^ ^35^ ^,^ ^36^ ^)^ A causa não é apenas a miopatia sarcoide, mas também os altos níveis circulantes de citocinas inflamatórias como TNF-α, IL-6 e IFN-γ.

Na miopatia sarcoide, três padrões clínicos são geralmente reconhecidos^(^ ^37^ ^)^: miopatia crônica (vista em 86% dos casos), que é a forma mais comum, caracterizada por um início insidioso de fraqueza muscular proximal com níveis séricos de enzimas musculares normais ou elevados; miosite aguda (vista em 11% dos casos); e miosite nodular ou tumoral (vista em apenas 3% dos casos). A miopatia nodular se manifesta como múltiplos nódulos palpáveis e tumorais nos músculos.^(^ ^37^ ^,^ ^38^ ^)^ O uso da RM e da PET/TC facilita o diagnóstico de sarcoidose muscular.

A base do tratamento de pacientes com sarcoidose muscular são 8-12 semanas de terapia com glicocorticoide sistêmico na dose diária inicial de 0,5-1 mg/kg, com redução progressiva. O metotrexato, a cloroquina e a azatioprina têm sido usadas em formas resistentes ao corticosteroide ou corticosteroide dependentes.^(^ ^39^ ^)^ A talidomida e o infliximabe se mostraram benéficos em alguns casos de miopatia sarcoide. A eficácia desses medicamentos parece estar relacionada à inibição do TNF-α. A miopatia induzida por corticosteroide também pode ocorrer como complicação do tratamento da sarcoidose. Os pacientes afetados tipicamente desenvolvem fraqueza muscular proximal de início gradual (ao longo de várias semanas) e acompanhada de perda muscular. Uma manifestação frequente é a dificuldade de se levantar de uma cadeira ou subir escadas. Mialgia e sensibilidade muscular não são observadas.^(^ ^40^ ^)^

## Combinação de sarcoidose e doença reumática

A sarcoidose pode estar associada a outras doenças inflamatórias crônicas como o lúpus eritematoso sistêmico (LES), a síndrome de Sjögren ou a artrite psoriásica. Na sarcoidose, a suspeita de LES é levantada quando o paciente desenvolve erupção em borboleta ou lesões discoides. O tratamento de pacientes com sarcoidose e LES é desafiador e deve ser individualizado.^(^ ^41^ ^)^ O uso de agentes anti-TNF-α deve ser evitado em pacientes que têm LES ativo. Em raros casos, a sarcoidose e a síndrome de Sjögren podem ambas afetar as glândulas salivares. Secura e edema difuso dos tecidos das mucosas orais pode ser o sintoma de apresentação da sarcoidose.^(^ ^42^ ^)^

Aproximadamente 6% de todos os pacientes com sarcoidose desenvolvem artrite psoriásica.^(^ ^43^ ^)^ Embora a terapia anti-TNF-α seja útil na psoríase, paradoxalmente, também pode induzir psoríase progressiva, e pacientes tratados com agentes anti-TNF-α devem ser monitorados cuidadosamente.

## Alterações no metabolismo do cálcio

Na sarcoidose, a hipercalciúria é mais frequente do que a hipercalcemia. Ambas podem ser causadas por nefrocalcinose, pedras nos rins ou insuficiência renal. Macrófagos granulomatosos aumentam a conversão da 25-hidroxivitamina D em 1,25-hidroxivitamina D (calcitriol), levando ao aumento da absorção de cálcio pelo intestino.^(^ ^44^ ^)^ Os altos níveis de calcitriol induzem a ativação de osteoclastos e a reabsorção óssea. Além disso, pacientes tratados com corticosteroides apresentam um maior risco de osteoporose. A perda óssea poderia ser também aumentada pelos altos níveis de peptídeo relacionado ao hormônio paratireoide identificados em tecido sarcoide.^(^ ^45^ ^)^ Os corticosteroides têm sido usados com sucesso para melhorar os distúrbios do metabolismo do cálcio.^(^ ^46^ ^)^ O micofenolato de mofetil e o infliximabe têm sido usados em casos selecionados.^(^ ^47^ ^,^ ^48^ ^)^

## Perda óssea na sarcoidose

A perda óssea na sarcoidose pode ser causada por vários fatores, incluindo granulomatose esquelética difusa, calcitriol, fator ativador de osteoclastos, e terapia com glicocorticoides, particularmente em pacientes na pós-menopausa. Em um estudo sobre corticoterapia em pacientes com sarcoidose, os autores observaram que a taxa de perda óssea em pacientes portadoras de sarcoidose na pós-menopausa que receberam corticosteroide foi maior do que a relatada para pacientes portadores de artrite reumatoide ou asma que receberam corticosteroide.^(^ ^49^ ^)^

A prevenção e o tratamento da perda óssea em pacientes com sarcoidose é difícil. O cálcio e a vitamina D, ambos frequentemente administrados a pacientes com risco de osteoporose, devem ser considerados com cautela em pacientes com hipercalcemia, hipercalciúria, níveis elevados de paratormônio e pedras nos rins.^(^ ^50^ ^)^ As diretrizes publicadas pelo *American College of Rheumatology* recomendam que pacientes que recebem corticosteroides devem ser submetidos a testes de densidade mineral óssea.^(^ ^51^ ^)^ A ferramenta de avaliação de risco de fratura da Organização Mundial da Saúde pode ser usada para calcular o risco de fratura do paciente.

Para pacientes com sarcoidose que apresentam risco de desenvolver osteoporose em razão do uso prolongado de corticosteroides, os bifosfonados são eficazes na prevenção de perda óssea induzida por glicocorticoides. O *American College of Rheumatology* recomenda o uso de bifosfonados em pacientes de baixo risco que recebem corticosteroides em doses = 7,5 mg/dia, assim como em todos os pacientes de médio ou alto risco que recebem corticosteroides. Dos bifosfonados disponíveis, o alendronato, o risedronato, o ácido zoledrônico e a teriparatida reduzem eficazmente a perda óssea e, assim, podem diminuir o risco de fratura.^(^ ^51^ ^)^ São necessários mais estudos sobre o uso de bifosfonados na osteoporose durante a sarcoidose.

## Considerações finais

Embora a sarcoidose possa afetar qualquer órgão, o acometimento do sistema músculo-esquelético é raro. A doença pode afetar os músculos, as articulações e os ossos. Essas condições, que são polimórficas, podem ser os sintomas de apresentação da doença ou podem aparecer no curso de sua progressão. Os corticosteroides são o pilar do tratamento da sarcoidose, mas apenas têm efeito de adiamento. São necessários ensaios prospectivos, randomizados e controlados para a avaliação de sua eficácia em casos de sarcoidose com complicações reumáticas.
